# Multidisciplinary care for tracheostomy patients: a systematic review

**DOI:** 10.1186/cc8159

**Published:** 2009-11-06

**Authors:** Marie Garrubba, Tari Turner, Clare Grieveson

**Affiliations:** 1 Centre for Clinical Effectiveness, Southern Health, Locked Bag 29, Clayton, Victoria 3168, Australia; 2 Monash Medical Centre, Speech Pathology, Southern Health, Locked Bag 29, Clayton, Victoria 3168, Australia

## Abstract

**Introduction:**

Appropriate care for patients with tracheostomies in hospital settings is an important issue. Each year more than 7000 patients receive tracheostomies in Australia and New Zealand alone. Many of these tracheostomy patients commence their care in the intensive care unit (ICU) and once stabilised are then transferred to a general ward. Insufficient skills and experience of staff caring for tracheostomy patients may lead to sub-optimal care and increased morbidity. The purpose of this review was to identify whether multidisciplinary tracheostomy outreach teams enable the reduction in time to decannulation and length of stay in acute and sub-acute settings, improve quality of care or decrease adverse events for patients with a tracheostomy.

**Methods:**

We included all relevant trials published in English. We searched Medline, CINAHL, All EBM and EMBASE in June 2009. Studies were selected and appraised by two reviewers in consultation with colleagues, using inclusion, exclusion and appraisal criteria established a priori.

**Results:**

Three studies were identified which met the study selection criteria. All were cohort studies with historical controls. All studies included adult patients with tracheostomies. One study was conducted in the UK and the other two in Australia. Risk of bias was moderate to high in all studies. All papers concluded that the introduction of multidisciplinary care reduces the average time to decannulation for tracheostomy patients discharged from the ICU. Two papers also reported that multidisciplinary care reduced the overall length of stay in hospital as well as the length of stay following ICU discharge.

**Conclusions:**

In the papers we appraised, patients with a tracheostomy tube in situ discharged from an ICU to a general ward who received care from a dedicated multidisciplinary team as compared with standard care showed reductions in time to decannulation, length of stay and adverse events. Impacts on quality of care were not reported.

These results should be interpreted with caution due to the methodological weaknesses in the historical control studies.

## Introduction

Appropriate care for patients with tracheostomies in hospital settings is an important issue. Each year more than 7000 patients receive tracheostomies in Australia and New Zealand alone [1]. Many of these tracheostomy patients commence their care in the intensive care unit (ICU) and once stabilised are transferred to a general ward. Insufficient skills and experience of staff caring for tracheostomy patients may lead to suboptimal care and increased morbidity.

To facilitate the improvement of care of patients with tracheostomy, Southern Health, Clayton, Victoria, Australia, is interested in planning a multidisciplinary outreach service to care for tracheostomy patients discharged from the ICU to the wards. To inform this process the Centre for Clinical Effectiveness was requested to undertake a systematic review to identify whether or not multidisciplinary tracheostomy outreach teams compared with standard care enable the reduction in time to decannulation and length of stay in acute and sub-acute settings, improve quality of care or decrease adverse events for these patients.

## Materials and methods

### Search strategy

In June 2009, we conducted a search for any comparative study written in English from 1980 onwards. We searched Medline using the following search strategy: (exp Tracheostomy/OR exp Tracheotomy/OR (tracheostom$ OR tracheotom$).mp. OR (trachea AND stoma).mp.) AND ((exp Patient Care Team/OR "patient care team".mp.) OR exp "Continuity of Patient Care"/OR exp Patient Care Planning/OR exp Case Management/OR exp Patient Care Management/OR exp "Delivery of Health Care, Integrated"/OR exp Patient-Centered Care/OR (Case-management OR care-coordination OR care-co-ordination OR care-planning).mp. OR (Multidisciplin$ OR multi-disciplin$ OR multiprofessional OR multi-professional OR interdisciplin$ OR inter-disciplin$ OR (multi$ AND profession$)).mp. OR (team$ OR service$).mp.)

Similar terms appropriately translated were used in EMBASE, All EBM and CINAHL. Studies were selected and appraised by two reviewers in consultation with colleagues using study selection and appraisal criteria established *a priori*.

### Inclusion criteria

The following inclusion criteria were applied to all studies identified.

Patient group included all tracheostomy patients, adults and/or children, from any age group, in a hospital ward setting. Intervention was multidisciplinary care. Comparator was standard care. Outcomes were average time to decannulation, length of stay, quality of care, and adverse events.

### Quality assessment

The quality of included cohort studies was appraised using the standard critical appraisal questions developed by the Centre for Clinical Effectiveness. Critical appraisal questions are outlined in Table 1.

**Table 1:** Critical appraisal questions for a cohort study

Description of the study
1. Patient/population
2. Number
3. Setting
4. Intervention
5. Comparison/control
6. Outcomes
7. Inclusion criteria
8. Exclusion criteria
Study validity
1. Were there any conflicts of interest in the writing or funding of this study?
2. Does the study have a clearly focused question?
3. Is a cohort study the appropriate method to answer this question?
4. Does the study have specified inclusion/exclusion criteria?
5. If there were specified inclusion/exclusion criteria, were these appropriate?
6. Other than the exposure under investigation, were the groups selected from similar populations?
7. Aside from the exposure, were the groups treated the same?
8. Was exposure measured in a standard, valid and reliable way?
9. Were outcome assessors blind to the exposure?
10. Were all outcomes measured in a standard, valid and reliable way?
11. Were outcomes assessed objectively and independently?
12. Is the paper free of selective outcome reporting?
13. Were the outcomes measured appropriate?
14. Was there sufficient duration of follow up?
15. Was the study sufficiently powered to detect any differences between the groups?
16. If statistical analysis was undertaken, was this appropriate?
17. Were the groups similar at baseline with regards to key prognostic variables?
18. What percentage of the individuals recruited into each arm of the study were lost to follow up?
19. What percentages of the individuals were not included in the analysis?
Other
1. What is the overall risk of bias?
Results
Authors' conclusions
Our comments

### Missing data

Authors of included studies were contacted by email with any queries.

## Results

### Search results

**Figure 1:**
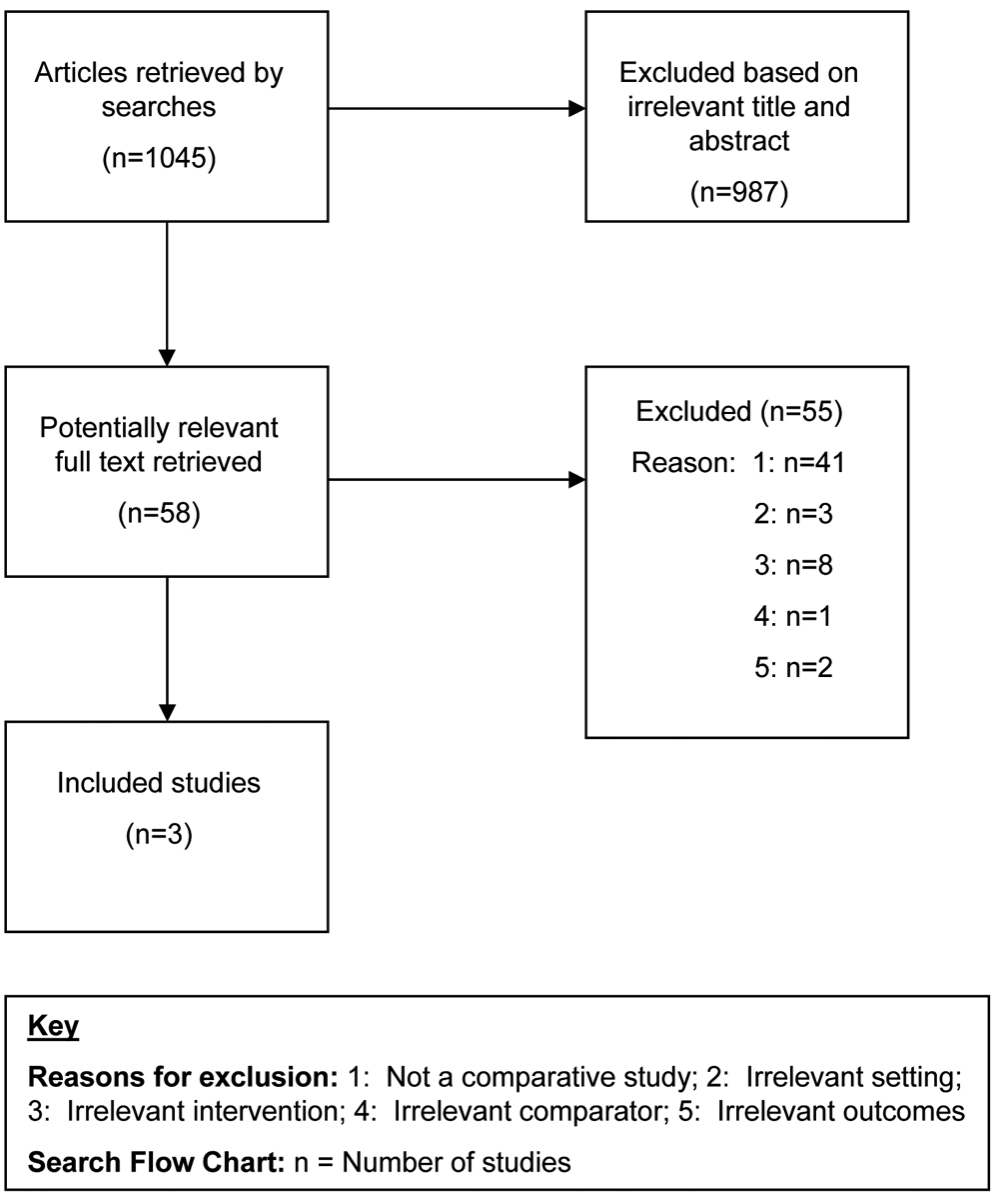
Number of studies included. Key reasons for exclusion: 1 = not a comparative study; 2 = irrelevant setting; 3 = irrelevant intervention; 4 = irrelevant comparator; 5 = irrelevant outcomes. Search flow chart: n = number of studies.

The search of all databases returned 1045 articles, which were reviewed by title and abstract. When a decision could not be made based on abstract alone, full text was retrieved. A total of 59 full text articles were retrieved for review and three articles met the inclusion criteria (Figure 1).

Three relevant studies were identified that met the study selection criteria. All were cohort studies with historical controls [2-4]. Critical appraisals of the quality of the three cohort studies are available [See additional data file 1].

All studies included adult patients with tracheostomies. One study was conducted in the UK [2] and the other two in Australia [3,4].

### Study results

The first study was a historical cohort study including patients with a tracheostomy discharged from an intensive treatment unit (ITU) to a general ward at St. Mary's Hospital, Paddington, London, UK. A total of 89 patients were included, of which 79 were the control group and 10 received the intervention. The intervention included a weekly Tracheostomy Multidisciplinary Team (TMDT) ward round (TMDT members included an ear nose and throat Specialist Registrar (ENT SpR) and Specialist Trainee Year 2 (ST2), speech and language therapist, respiratory physiotherapist and a critical care outreach nurse), monthly teaching sessions organised for nursing staff involved with tracheostomy care and an ENT-led training day for physiotherapists and speech and language therapists. This intervention was compared retrospectively with standard care. The study looked at the impact of the intervention on the following outcomes: time to tracheostomy tube decannulation post-ITU discharge, total time of tracheostomy (not defined, but we can presume the definition of total time is inclusive of ITU and general ward stay) and compliance with local tracheostomy care guidelines (St. Mary's tracheostomy care bundle) between the intervention group and a group of 70 patients of whom little information is provided for selection criteria (this outcome was therefore excluded from the appraisal of this paper).

The methods of this study were not well documented. Overall we found the risk of bias in this study to be high. Inclusion and exclusion criteria were not clearly documented; group similarity was not achieved (eg 10-year mean age difference); measurement of exposure and outcomes was not standardised, valid or reliable; and there was some uncertainty about the percentage lost to follow up. Contributing to the high risk of bias is the historical control study design. A historical control produces opportunities for bias, which can arise from the dissimilarity between control and treatment groups, differences between the hospital environment at the time of the intervention and earlier conditions at the time of the historical control and the difficulty in controlling for confounding.

The study found that "The mean time to decannulation following ITU discharge was significantly reduced from 21 to 5 days (*P *= 0.0005)" and that "The total tracheostomy time was reduced from 34 to 24 days, although this difference was not statistically significant (*P *= 0.13)".

The second study was also a historical cohort study including ICU patients not under the care of an ENT unit who were discharged to the ward with a tracheostomy at St. Vincent's Hospital, Melbourne, Australia. A total of 280 patients were included in the study of which 41 were the control group and 239 received the intervention over three years. The intervention in this study was an intensivist-led, multidisciplinary team consisting of an intensivist, an ICU liaison nurse, a physiotherapist, a speech pathologist and a dietitian. This team undertook twice weekly ward rounds to review patients and to plan and oversee an individualised tracheostomy weaning programme. The intervention patients were compared with patients who received standard care by a physiotherapist and speech pathologist with *ad hoc *input from doctors prior to the introduction of the intervention. The standard care in this study appears to be multidisciplinary, but is not a dedicated service. The study looked at decannulation time from ICU discharge as its primary outcome and hospital length of stay, length of stay after ICU discharge and length of stay of less than 43 days (the upper trim point for the disease-related group code for tracheostomy) as secondary outcomes.

The methods of this study were fairly well documented, but some methodological weaknesses may affect the conclusions of the study. The study presents patient groups chosen from similar populations; however, the difference in the size of the control versus intervention group is substantial (n = 41 and n = 239, respectively) and the larger group may include a wider range of patient types. Both groups were reportedly treated the same with the exception of the patients in the third year (2006) of the intervention group whose results may have been affected by the introduction of a nurse liaison service. Despite the study reporting similar populations and treatment of patients between the control and intervention groups, the historical control study design introduces the potential for unknown confounders and historical factors that may have affected the results.

It appears that measurement of outcomes were not standardised, valid or reliable. There was no loss to follow up with all patients being included in the final analysis.

The study had three findings. (1) The median hospital length of stay decreased over the study period from 42 (interquartile range (IQR): 29 to 73) days to 34.5 (IQR: 26 to 53) days (*P *= 0.06). (2) The median hospital stay after ICU discharge was reduced in 2006 compared with 2003, from 30 (IQR: 13 to 52) days to 19 (IQR: 10 to 34) days (*P *< 0.05). This data was provided for the comparison between 2006 and 2003 only. Statistical significance was not reported for other intervention years. (3) There was a significant trend to reduced decannulation times from ICU discharge (*P *< 0.01) across the four years of the study, although absolute difference between the years was not statistically significant (*P *= 0.06).

The third study was a historical cohort study (matched pairs design) including spinal cord injury (SCI) patients with a tracheostomy tube *in-situ *discharged to wards at the Austin Hospital, Melbourne, Australia. A total of 34 patients were recruited and analysed in the pre-Tracheostomy Review and Management Service (TRAMS) arm of the study, while 53 patients were recruited to the post-TRAMS arm. Of the 53 patients, 34 were matched by level of SCI, injury severity and age to the controls (pre-TRAMS) and included in the analysis. The intervention was a TRAMS introduced as a consultative team of respiratory and ICU doctors, clinical nurse consultants, physiotherapists and speech pathologists. The service included: twice weekly ward rounds by the TRAMS team for all ward-based patients with a tracheostomy tube (except ENT in-patients); patient consultations on other days as needed; patient support, and education of ward staff; regular assessment of patient readiness for decannulation; support of patients with a long-term tracheostomies in the community, with equipment, consumables, tube changes and education; tracheostomy resource and equipment library; implementation and review of interdisciplinary tracheostomy policy and procedures; critical incident review and delivery of interdisciplinary tracheostomy education. This intervention was compared with pre-TRAMS care within the Victorian Spinal Cord Service at Austin Health. The study looked at the impact of TRAMS on the following outcomes: length of acute hospital stay, duration of cannulation, improved communication through use of one-way valve, adverse events and related costs.

The methods of this study were fairly well documented. Overall, we found the risk of bias in this study to be moderate.

The authors did not report any conflicts of interest in the writing or funding of the study or whether outcomes were measured in a standard, valid and reliable way. It was also unclear if outcomes were assessed objectively and independently, and if participants had sufficient duration of follow up. A large percentage of patients (46%) from the post-TRAMS group were excluded from the analysis as well as 13% from the pre-TRAMS group (See details in Additional data file 1). Contributing to the moderate risk of bias is the lack of information provided for the methods of matching the post-TRAM patients to the pre-TRAM patients. The lack of information around the method of matching opens this paper to potential bias because it is unclear whether the researchers could influence the choice of patients to be matched. Similarly with all historical cohort studies there is a possibility that other factors may have influenced the tracheostomy care of SCI patients either positively or negatively.

The study found that the median length of acute hospital stay was reduced from 60 to 41.5 days (*P *= 0.03) with the median duration of cannulation also reduced from 22.5 to 16.5 days (*P *= 0.03). These results were both statistically significant. The post-TRAMS group reported no adverse events as compared with the two tracheostomy related code-blue calls for the pre-TRAMS group.

## Discussion

All papers included in this review came to the conclusion that the introduction of multidisciplinary care reduces the average time to decannulation for tracheostomy patients discharged from the ICU to a general ward setting. Two papers [3,4] also reported that multidisciplinary care reduced the overall length of stay in hospital, as well as the length of stay from ICU discharge. Although these results are encouraging, the historical control design presents a significant potential for bias in all studies. Studies designed around a historical control are open to bias from many angles. The dissimilarities between the control and treatment group, whether demographic, diagnostic criteria, stage and severity of disease, simultaneous treatments, and differences in observational and data collection conditions, can affect outcomes. Similarly, the time difference between control and intervention groups can introduce differences other than the intervention; for example, change in treatment patterns (eg protocols, guidelines, and changes in staffing) and other exposures that are unknown to data collectors or not recorded in medical records. All of these variables have the potential to affect the results of the studies appraised.

Multidisciplinary care is a complex intervention that is difficult to evaluate due to its multiple and varying components. All appraised studies presented different descriptions of multidisciplinary care including different collaborations of disciplines. Therefore, it is difficult to infer the combination of disciplines that should make up the most appropriate multidisciplinary care team for tracheostomy patients.

It should be noted that in these studies [2-4] the multidisciplinary teams were led by different specialists: an intensivist, an ENT specialist and a respiratory physician, respectively. This is important because it may limit the generalisability of multidisciplinary teams for tracheostomy care as we are unable to tell whether the effects reported were due to the dedicated 'tracheostomy' feature, the multidisciplinary nature of the care or the medical and specialist nature of the leadership.

Multidisciplinary tracheostomy teams are now widespread in national and international health services and are seen to be the most appropriate model of care for tracheostomy patients [2-7]. This review suggests that although there is some evidence that a specialised, multidisciplinary tracheostomy team may be beneficial; however, this evidence is limited and high-quality evidence from well-controlled studies including data on complication rates and adverse events is still needed to convincingly determine the effectiveness of a multidisciplinary team for tracheostomy patients.

Given the potential for bias in the studies reviewed the results should be interpreted with care.

## Conclusions

In the papers we appraised, patients with a tracheostomy tube *in situ *discharged from an ICU to a general ward who received care from a dedicated multidisciplinary team as compared with standard care showed improvements in time to decannulation, length of stay and adverse events. The effects of the intervention on quality of care were not reported. These results may be applicable to the Southern Health setting; however, should be actioned with caution due to the methodological weaknesses presented in the historical control studies.

## Key messages

• Multidisciplinary tracheostomy teams are now widespread in national and international health services and are seen to be the most appropriate model of care for tracheostomy patients.

• High-quality evidence from well-controlled studies is still needed to convincingly determine the effectiveness of a multidisciplinary team for tracheostomy patients.

• All papers included in this review came to the conclusion that the introduction of multidisciplinary care reduces the average time to decannulation for tracheostomy patients discharged from the ICU to a general ward setting.

• Two papers reported that multidisciplinary care reduced the overall length of stay in hospital as well as the length of stay from ICU discharge.

• Generalisability of multidisciplinary teams for tracheostomy care is limited as all three teams were led by different specialists; an intensivist, an ENT specialist and a respiratory physician.

## Abbreviations

ENT: ear, nose and throat; ICU: intensive care unit; ITU: intensive treatment unit/intensive therapy unit; IQR: interquartile range; SCI: spinal cord injury; SpR: specialist registrar; ST2: specialist trainee year 2; TMDT: tracheostomy multidisciplinary team; TRAMS: tracheostomy review and management service.

## Competing interests

The authors declare that they have no competing interests.

## Authors' contributions

CG requested the systematic review from the Centre for Clinical Effectiveness and provided clinical expertise and interpretation. With assistance from CG, MG and TT developed the search strategy. MG applied inclusion criteria to search results in consultation with TT. MG appraised the three included papers. TT was a second review for all included papers. MG prepared the first draft of this article which TT and CG then reviewed.

## Authors' information

MG is a Clinical Effectiveness Project Officer at the Centre for Clinical Effectiveness, Southern Health. TT is a Clinical Effectiveness Senior Consultant at the Centre for Clinical Effectiveness, Southern Health. CG is the Manager of Speech Pathology at Southern Health.

## Supplementary Material

Additional file 1 A pdf file containing the critical appraisal tables of all three included studies. Click here for file
