# Supplementary material for: Multidisciplinary care for tracheostomy patients: a systematic review
Source: Crit Care. 2009 Nov 6;13(6):R177. doi: 10.1186/cc8159 (PMC2811928; doi:10.1186/cc8159)
Supplement: Additional file 1 — A pdf file containing the critical appraisal tables of all three included studies. [file cc8159-S1.PDF]

Additional Data File 1 – Critical Appraisal Tables

Study: Arora A, Hettige R, Ifeacho S and Narula A. (2008) Driving standards in tracheostomy care: a preliminary communication of the St Mary's ENT-led multidisciplinary team approach. *Clin. Otolaryngol*, **33**: 596-599

Description of study

**Patient/population**	Patients with a tracheostomy discharged from ITU (ITU not defined but likely to be "Intensive Treatment Unit" or "Intensive Therapy Unit") to general wards
**N**	Control = 79 of 148 analysed, Intervention = 10<br>Data was also reported on an additional 70 patients for compliance to the components of the tracheostomy care bundle. No information was provided for how these patients were selected and it is unclear if these 70 patients were a subset of the 79 analysed.
**Setting**	Tertiary academic London hospital serving an inner city population of multi-ethnic background (St Mary's Hospital, Paddington, London)
**Intervention**	▪ Introduction of a weekly Tracheostomy Multidisciplinary Team (TMDT) ward round (TMDT members included an ENT SpR and ST2 (SpR and ST2 were not defined but are likely to be "Specialty Registrar and "Specialty Trainer"), speech and language therapist, respiratory physiotherapist and critical care outreach nurse).<br>▪ Monthly teaching sessions organised for nursing staff involved with tracheostomy care<br>▪ ENT-led training day for physiotherapists and speech and language therapists
**Comparison/control**	Standard care
**Outcomes**	▪ Compliance with local tracheostomy care guidelines (St. Mary's tracheostomy care bundle) – not relevant to this review as compliance was compared between the intervention group and another group of seventy patients for whom little information was provided regarding selection criteria.<br>▪ Time to tracheostomy tube decannulation – post ITU discharge<br>▪ Total time of tracheostomy – not defined however we can presume definition of total time is inclusive of ITU and general ward stay
**Inclusion Criteria**	No specific inclusion criteria reported however for both control and intervention groups only patients with a tracheostomy discharged from ITU to a general ward were included.
**Exclusion Criteria**	No specific exclusion criteria reported however, study does report that 69 patients were excluded from the 148 historical controls due to death during tracheostomy management, transfer to another trust or missing data regarding tracheostomy status.

Study Validity

**Were there any conflicts of interest in the writing or funding of this study?**	No	The authors declared they had no conflicts of interest.
**Does the study have a clearly focused question?**	Yes	The population studied, intervention, comparators and outcomes considered were all clearly described.
**Is a cohort study the appropriate method to answer this question?**	Partial	A cohort study is an appropriate way of answering the question however an RCT would be less open to bias.
**Does the study have specified inclusion/exclusion criteria?**	No	It was unclear whether inclusion or exclusion criteria were established *a priori*.<br>The study explains that 148 patients were treated during the control period and of these 69 were excluded for reasons related to death, transfer to another trust or missing data regarding tracheostomy status

**If there were specified inclusion/ exclusion criteria, were these appropriate?**	N/A	
**Other than the exposure under investigation, were the groups selected from similar populations?**	No	Although the groups were selected from the same population (St.Mary's Hospital) the study is a historical cohort which introduces bias. We cannot be sure that other variables introduced between the control and intervention periods have not influenced the outcomes for example; introduction of new protocols of care, change in staff etc could have contributed to reduced time to decannulation.<br>The authors report a large difference in patient numbers included in the control and intervention groups (n=79 for control, n=10 for intervention). The authors justify this by stating *"The TMDT and other implemented changes are set to continue which will enable data collection from a larger cohort of patients". (p599)*
**Aside from the exposure, were the groups treated the same?**	Not reported	It was not reported whether the groups were treated the same. As the control group in this study is a historical cohort we cannot be sure that other factors, such as change in staff or a change in care protocols, were not responsible for the effect reported.
**Was exposure measured in a standard, valid and reliable way?**	Not reported	The authors did not report whether the intervention was measured in a standard, valid or reliable way. Therefore, we can not be sure that all patients in the intervention group received the intervention or all patients in the historical cohort did not receive a form of TMDT care.
**Were outcome assessors blind to the exposure?**	No	The outcome assessors were not blind to the exposure (care by a TMDT).
**Were all outcomes measured in a standard, valid and reliable way?**	Not reported	The authors did not report whether the outcomes were measured in a standard, valid and reliable way. For the outcome we are interested in (time to decannulation) we cannot be sure what the time recorded refers to, for example, it could be the time the decannulation is ordered as opposed to the time it is performed.
**Were outcomes assessed objectively and independently?**	Not reported	The authors did not report whether the outcomes were measured objectively.
**Is the paper free of selective outcome reporting?**	No	As there is no published protocol it is not clear if all planned outcomes were measured and reported.<br>Although not included as a main outcome measure in the methods the authors reported total tracheostomy time as an additional outcome.
**Were the outcomes measured appropriate?**	Yes	
**Was there sufficient duration of follow-up?**	Not reported	It is unclear if each of the patients was followed up long enough to see the effects of the intervention after decannulation.
**Was the study sufficiently powered to detect any differences between the groups?**	Not reported	The authors do not discuss which outcomes the study was powered for.<br>For the outcome we are interested in 'time to decannulation' the authors reported a significantly reduced time to decannulation of 21 to 5 days (p=0.0005) which demonstrates that appropriate power was achieved.<br><br>The authors also report 'total time of tracheostomy' which although not defined we presume includes time of tracheostomy placement in ITU to decannulation in a general ward. They also report a reduction in total time of tracheostomy of 34 days to 24 days. This result is not statistically significant p=0.13 however may be clinically significant.

**If statistical analysis was undertaken, was this appropriate?**	Partial	For the outcome we are interested in 'time to decannulation' the authors performed a *Mann Whitney Wilcoxon Test*.<br>It is unclear whether the statistical analysis was planned *a priori* or if the data was analysed according to the study protocol. Similarly we are unsure if the data has been checked for normality.<br>No measures of variability are presented for the outcomes.<br>There does not appear to be any missing data.
**Were the groups similar at baseline with regards to key prognostic variables?**	Partial	Groups were similar in their APACHE II mean scores, however a ten year difference in mean age and lack of reporting of data on sex of participants and type of illness may mean there were clinically important differences between the groups at baseline.
**What percentage of the individuals recruited into each arm of the study were lost to follow-up?**	0% exposed<br>unclear % control	Sixty-nine of the 148 control participants were excluded from the study. Reasons for exclusion were death, transfer to another trust, or no information available in patient record for tracheostomy status. It is unclear whether or not these patients should have been included.
**What percentages of the individuals were not included in the analysis?**	0% exposed<br>unclear % control	All patients in the exposed group were included in the analysis.<br>Sixty-nine of the 148 patients with tracheostomy excluded from the study were not included in the analysis

Other

**What is the overall risk of bias?**	High	Few criteria for methodological quality of cohort studies were fulfilled therefore it is unknown if the conclusions made regarding time to decannulation have been affected, however the apparent weaknesses in design and the historical cohort method make bias likely.

Results

<u>Time to decannulation</u>

"The mean time to decannulation following ITU discharge was significantly reduced from 21 to 5 days ($p = 0.0005$)".

The study also reported on compliance with nursing care standards and total time tracheostomy was in situ which was not part of our question.

Author's Conclusions

"The introduction of regular ENT-led multidisciplinary input for patients with a tracheostomy significantly improved compliance with nursing care standards. There was also a reduction in the total length of time tracheostomy tubes remain in situ, with time to decannulation significantly reduced." (Abstract)

Our comments

The authors of this paper suggest that time to decannulation is greatly reduced with the introduction of a regular ENT-led multidisciplinary care team. This is a reasonable conclusion of clinical significance; however the reliability of this result is questionable as the methods are not well documented. It is unclear whether the historical cohort design, which presents a significant potential for bias, has affected the outcomes observed.

Study: Tobin AE and Stantamaria JD. (2007) An intensivist-led tracheostomy review team is associated with shorter decannulation time and length of stay: a prospective cohort study. *Critical Care*, **12**: R48

Description of study

**Patient/population**	Intensive Care Unit (ICU) patients not under the care of an Ear, Nose and Throat (ENT) unit who were discharged to the ward with a tracheostomy
**N**	Control = 41, Intervention = 239
**Setting**	St. Vincent's Hospital, Melbourne, Australia (Tertiary Academic Hospital)
**Intervention (exposure)**	During normal working hours: Intensivist-led multidisciplinary team consisting of an Intensivist, an ICU liaison nurse, a physiotherapist, a speech pathologist and a dietician.<br>During out of hours: ICU provides any necessary assistance for acute problems either by direct consultation or via the Medical Emergency Team (MET)/cardiac arrest teams that are run in conjunction with the ICU.
**Comparison/control**	Standard care which included oversight of weaning of all patients not under the ENT unit bed care by a physiotherapist and speech pathologist with ad hoc input from doctors.
**Outcomes**	Primary outcome: decannulation time from ICU discharge<br>Secondary Outcome: hospital length of stay, length of stay after ICU discharge and length of stay of less than 43 days (the upper trim point for the disease related group [DRG] code for tracheostomy).
**Inclusion Criteria**	Control: Patients in the ICU database who had a tracheostomy while in ICU in 2003<br>Intervention: All tracheostomy patients discharged from the ICU alive who were not under the ENT unit's care during 2004 to 2006.
**Exclusion Criteria**	Control: not reported<br>Intervention: not reported

Study Validity

**Were there any conflicts of interest in the writing or funding of this study?**	No	The authors declared that they had no conflicts of interest.
**Does the study have a clearly focused question?**	Yes	The population studied, intervention, comparators and outcomes considered were all clearly described.
**Is a cohort study the appropriate method to answer this question?**	Partial	A cohort study is an appropriate way of answering the question however an RCT would be less open to bias.
**Does the study have specified inclusion/exclusion criteria?**	Partial	The study mentions criteria for including patients but does not provide any specific exclusion criteria. It seems likely that the inclusion of patients was defined *a priori.*
**If there were specified inclusion/ exclusion criteria, were these appropriate?**	Yes	The criteria noted for including patients into the study were appropriate.
**Other than the exposure under investigation, were the groups selected from similar populations?**	Partial	The two groups in this study were selected from St.Vincent's Hospital, Melbourne.<br>The authors discuss the 'major limitation' of the study as being the retrospective nature of the data collection. Controls were selected from a historical cohort and the authors accept that there may have been 'other factors influencing tracheostomy care – either positively or negatively'.

**Aside from the exposure, were the groups treated the same?**	Partial	The authors highlight that the Medical Emergency Team (MET) system was in place for both periods, suggesting that this is unlikely to be a factor that has affected the results. The authors also state that an ICU liaison nurse service began in 2006 which may have had an impact on the results. It may also be likely that general staff changes during the time between control and intervention could have affected the treatment of groups.
**Was exposure measured in a standard, valid and reliable way?**	Partial	Exposure to care by a multidisciplinary team for tracheostomy patients is likely to have been measured in a standard and reliable way. Introduced as a new method of care from 2004 onwards, the only inconsistency would be care of out of hours patients who were seen to by direct consultation or via MET/cardiac arrest teams run in conjunction with the ICU.
**Were outcome assessors blind to the exposure?**	No	The outcome assessors in this study were not blind to exposure.
**Were all outcomes measured in a standard, valid and reliable way?**	Not reported	The authors did not report whether the outcomes were measured in a standard, valid and reliable way.<br>For decannulation time we cannot be sure what the time recorded refers to, for example, it could be the time the decannulation is ordered as opposed to the time it is performed.
**Were outcomes assessed objectively and independently?**	Not reported	The authors did not report whether the outcomes were measured objectively.
**Is the paper free of selective outcome reporting?**	Partial	The authors report on primary and secondary outcomes. They also report on additional outcomes including ICU length of stay, time to tracheostomy, decannulation to discharge, decannulated or not decannulated and decannulated and less than 43 days. As there is no published protocol it is not clear if all planned outcomes were measured and reported.<br>The authors have only reported comparison of outcomes between the control data (2003) and the third year of the intervention (2006). The paper lacks analysis of the combined years of the intervention (2004, 2005 and 2006).
**Were the outcomes measured appropriate?**	Yes	
**Was there sufficient duration of follow-up?**	Not reported	It is unclear if each of the patients was followed up long enough to see the effects of the intervention.
**Was the study sufficiently powered to detect any differences between the groups?**	Partial	The authors do not report whether the outcomes of this study were powered to find an effect however, power was achieved in two of the outcomes as a statistically significant outcome was reported. It can not be determined if the third outcome was powered correctly as it did not produce a statistically significant result.<br>• Trend in LOS statistically significant $p<0.05$<br>• Reduced decannulation time $p<0.01$<br>• While it appeared to be a clinically important difference an absolute difference between years for decannulation time did not meet criteria for statistical significance (2003; 14(7-31) compared to 2006; 7(3-17), ($p<0.06$)).
**If statistical analysis was undertaken, was this appropriate?**	Yes	Statistical tests performed included:<br>• Univariate analyses – Kruskal-Wallis test for continuous variables and chi-square or Fisher's exact test for categorical variables.<br>• Trend over time was examined using Cuzick's test for trend for continuous variables and the chi-square trend test for categorical variables.<br>• Kaplan-Meier survival curves for decannulation times were compared with the log-rank test.<br>• Multivariable analysis of decannulation times was undertaken using a Cox proportional hazards model. The proportional hazards assumption was inspected graphically and tested statistically. Hazard ratios are presented with 95% confidence intervals. A *P* value of less than 0.05 was assumed to indicate statistical significance.<br>Analyses were performed with STATA version 9.2.

**Were the groups similar at baseline with regards to key prognostic variables?**	Yes	Differences between key prognostic variables of age, sex and disease severity are not significant. Control and intervention groups had similar mean ages, similar proportion of men to women and similar APACHE II scores.
**What percentage of the individuals recruited into each arm of the study were lost to follow-up?**	0% exposed<br>0% control	All patients were properly accounted for.
**What percentage of the individuals were not included in the analysis?**	0% exposed<br>0% control	All patients were included in the analysis of the data.

Other

**What is the overall risk of bias?**	Moderate	In this study, most of the criteria of methodological quality have been fulfilled. As this study is based on a historical cohort it is open to bias but it appears to be well carried out and reported.

Results

Hospital length of stay

The median hospital length of stay was lower in the intervention group (34.5 [IQR: 26 – 53] days to 42 [IQR: 29 – 73] days, p=0.06), but the difference was not statistically significant.

Length of stay after ICU

The median hospital stay after ICU discharge was lower in the 2006 intervention group 30 (IQR: 13 – 52) days compared to the 2003 control group 19 (IQR: 10 – 34) days, ($p<0.05$). Comparisons for other years were not provided.

Decannulation time

There was a significant trend to reduced decannulation times from ICU discharge 14 (IQR: 7 – 31) days to 7 (IQR: 3 – 17) days ($p<0.01$), over the duration of the intervention although absolute difference between the years did not meet the criteria for statistical significance (p = 0.06).

Other outcomes measured

The study reported on outcomes other than those that were of interest to the requestor:

- ICU length of stay
- Decannulation to discharge
- Decannulated
- Not decannulated
- Discharge <43 days
- Decannulated and discharged <43 days

Author's Conclusions

"The institution of a tracheostomy team to manage tracheostomy care of patients discharged from the ICU with a tracheostomy was associated with improvements in decannulation rates and in length of stay. As well as improving patient care, services such as this may result in cost savings for the health service."

Our comments

The authors of the paper suggest that patients managed by a tracheostomy care team are associated with improvements in decannulation rates and length of stay however, the differences seen were not statistically significant. The authors also conclude that a tracheostomy care team service may result in cost savings for the health service but have not documented any results to this effect. Generally, this study was well carried out and reported however it remains unclear whether the historical cohort design, which presents a significant potential for bias in this study and the introduction of a nurse liaison service in the third year of the intervention, has affected the outcomes observed.

Study: Cameron TS, McKinstry A, Burt SK, Howard ME, Bellomo R, Brown DJ, Ross JM, Sweeney JM and O'Donoghue FJ. 2009. Outcomes of patients with spinal cord injury before and after introduction of an interdisciplinary tracheostomy team. *Crit Care Resusc,* **11**: 14-19.

Description of study

**Patient/population**	Spinal cord injury (SCI) patients with a tracheostomy
**N**	Control = 34, Intervention = 34 (plus 19 potential participants who were not matched to patients from the control period)
**Setting**	The Austin Hospital, Melbourne Victoria (Tertiary Hospital)
**Intervention (exposure)**	Tracheostomy Review and Management Service (TRAMS) introduced as a consultative team of respiratory and ICU doctors, clinical nurse consultants, physiotherapists and speech pathologists.<br>Services included:<br>• Twice weekly ward rounds by the TRAMS team for all ward-based patients with a tracheostomy tube (except ear, nose and throat in-patients)<br>• Patient consultations on other days as needed<br>• Patient support, and education for ward staff<br>• Regular assessment of patient readiness for decannulation<br>• Support of patients with a long-term tracheostomies in the community, with equipment, consumables, tube changes and education<br>• Tracheostomy resource and equipment library<br>• Implementation and review of interdisciplinary tracheostomy policy and procedures<br>• Critical incident review<br>• Delivery of interdisciplinary tracheostomy education
**Comparison/control**	Pre TRAMS care within the Victorian Spinal Cord Service (VSCS) at Austin Health.
**Outcomes**	Comparison of length of acute hospital stay, duration of cannulation, improved communication through use of one-way valve, number of adverse events and related costs.
**Inclusion Criteria**	SCI patients requiring a tracheostomy
**Exclusion Criteria**	Several patients who met the inclusion criteria were excluded from the analysis based on the following criteria:<br>• Pre-TRAMS – 1 patient died of non tracheostomy related causes and 4 patients had a permanent tracheostomy.<br>• Post-TRAMS – 3 patients died of non tracheostomy related causes and 7 patients had a permanent tracheostomy<br>• Post-TRAMS – 19 of the 53 patients from the post-TRAMS group received the intervention but were not matched to the pre-TRAMS patients

Study Validity

**Were there any conflicts of interest in the writing or funding of this study?**	Not reported	The authors did not declare whether or not they had any conflicts of interest.<br>The manager of the TRAMS team is the lead author on this paper.
**Does the study have a clearly focused question?**	Yes	The population studied, intervention, comparators and outcomes considered were all clearly described.
**Is a cohort study the appropriate method to answer this question?**	Yes	A cohort study is an appropriate way of answering the question however an RCT would be less open to bias.
**Does the study have specified inclusion/exclusion criteria?**	Yes	The study mentions specific criteria for including and excluding patients. It appears that the inclusion and exclusion of patients was defined a priori.
**If there were specified inclusion/ exclusion criteria, were these appropriate?**	Yes	The criteria noted for including patients into the study were appropriate. Exclusion criteria were noted for excluding patients from the analysis rather than the intervention.
**Other than the exposure under investigation, were the groups selected from similar populations?**	Partial	The two groups in this study were selected from the Austin Hospital Melbourne.<br>The authors note that a 20-month interval was left between the two cohorts to minimise possible contamination effects during the period in which new practices were introduced. Patients in the intervention group were matched to patients in the control group within one level of SCI, one grade of injury severity and within 10 years of age. The level and severity of injury were assessed by the Victorian Spinal Cord Service (VSCS) at the Austin Hospital.
**Aside from the exposure, were the groups treated the same?**	Partial	The authors note that the pre-TRAMS and post-TRAMS (control and intervention) cohorts were managed at the same centre under the same primary SCI team.<br>The authors highlight that by matching patients by major confounding variables for the study outcomes bias should have been minimised however as with all historical cohort studies there is a possibility that other factors may have influenced SCI patient's tracheostomy care either positively or negatively (eg staff changes).
**Was exposure measured in a standard, valid and reliable way?**	Yes	Exposure to care by the TRAMS team for SCI patients with a tracheostomy tube is likely to have been measured in a standard and reliable way.
**Were outcome assessors blind to the exposure?**	Not reported	It is unclear if outcome assessors were blind to the exposure.
**Were all outcomes measured in a standard, valid and reliable way?**	Not reported	The authors did not report whether the outcomes were measured in a standard, valid and reliable way.<br>For duration of cannulation we cannot be sure what the end point of duration refers to, for example, it could be the point at which the decannulation is ordered as opposed to the time it is performed.<br>Adverse events measured included number of code-blue medical emergency calls and tracheostomy-related deaths. These outcomes are fairly objective however the criterion for deciding "tracheostomy-related" is prone to subjectivity.
**Were outcomes assessed objectively and independently?**	Not reported	The authors did not report whether the outcomes were measured objectively. However it is likely that the outcome measures were exposed to a level of subjectivity.
**Is the paper free of selective outcome reporting?**	Partial	The authors report on all of the identified main outcomes.<br>As there is no published protocol it is not clear if all planned outcomes were measured and reported.
**Were the outcomes measured appropriate?**	Yes	
**Was there sufficient duration of follow-up?**	Not reported	It is unclear if the patients were followed up long enough to see the effects of the intervention.

**Was the study sufficiently powered to detect any differences between the groups?**	Yes	The authors do not report whether the outcomes of this study were powered to find an effect however, power was achieved in the two outcomes of interest as a statistically significant outcome was reported.<br>• Reduction in acute hospital length of stay was statistically significant p=0.03<br>• Reduction in duration of cannulation was statistically significant p=0.03
**If statistical analysis was undertaken, was this appropriate?**	Yes	Statistical tests performed included:<br>• Wilcoxon sign-rank test – for assessment of the relationship between introduction of TRAMS program and length of stay, duration of cannulation and time to use of a one-way valve<br>• X^2 test – for assessment of the relationship between introduction of TRAMS and frequency of use of a one-way valve<br>• Kaplan-Meier survival analysis and log-rank test for equality of survival functions – to assess one-way valve use<br>Normality was assessed for all analyses, and appropriate non-parametric methods were applied as necessary using a matched-pairs analysis.<br>All data analysis was performed using Intercooled Stata version 9. Statistical significance levels were set at p<0.05.
**Were the groups similar at baseline with regards to key prognostic variables?**	Yes	Differences between age, sex, type of tracheostomy level of spinal injury and number of ICU hours were not statistically significant.
**What percentage of the individuals recruited into each arm of the study were lost to follow-up?**	0% exposed<br>0% control	All patients recruited into this study were accounted for. Clarification was sought from the corresponding author regarding the number of patients included in the post-TRAMS population. The author confirmed that there was only 34 patients in the post-TRAMS population and not 53 as noted in the figure 1 flow chart of patients in the study.
**What percentage of the individuals were not included in the analysis?**	46% exposed<br>13% control	Patients with a permanent tracheostomy and those that died from non-tracheostomy related causes were excluded from the analysis. A further 19 patients were excluded from the analysis as they were not matched to any patients in the pre-TRAMS group.<br><u>Control (Pre-TRAMS):</u><br>5 patients excluded from analysis (1 died, 4 had permanent tracheostomy) / 39 (included) = 13%<br><u>Exposed (Post-TRAMS):</u><br>29 patients excluded from analysis (3 died, 7 had permanent tracheostomy and 19 were not matched) / 63 = 46%

Other

**What is the overall risk of bias?**	Moderate	In this study, most of the criteria of methodological quality have been fulfilled. As this study is based on a historical cohort it is open to bias but it appears to be well carried out and reported.

Results

Length of stay

Median length of acute hospital stay was reduced from 60 days (IQR: 37.8 – 106.5) in the control group to 41.5 days (IQR: 28.75 – 61.75) in the exposed group, (p=0.03)

Duration of cannulation

Median duration of cannulation for the control group was 22.5 days (IQR 17 – 58) compared to 16.5 days (IQR: 12 – 25), in the exposed group (p=0.03).

Adverse events

Reported adverse events included two tracheotomy-related code blue calls for patients in the control group and none in the intervention group.

Other outcomes measured

The study reported on outcomes other than those that were of interest to the requestor:

- Communication through use of one-way valve
- Costs

Author's Conclusions

"Implementing a tracheostomy review and management service (TRAMS) was associated with improved patient outcomes for SCI patients with a tracheotomy. Tracheostomy tubes were removed earlier, with a significant reduction in acute hospital length of stay and substantial cost savings. More patients were able to speak significantly sooner than before the introduction of TRAMS. The incidence of adverse events remained low despite earlier removal of tracheostomy tubes."

Our comments

The authors of this paper suggest that the implementation of a tracheostomy review and management service (TRAMS) was associated with improved patient outcomes for SCI patients with a tracheostomy tube in situ. This is a reasonable conclusion supported by statistically significant results for reduced length of stay and duration of cannulation. Generally, this study was well carried out and reported however it remains unclear whether the historical cohort design, which presents a significant potential for bias in this study has affected the results. The exclusion of 19 patients as part of the matching process is not well documented. The lack of information around the method of matching opens this paper to potential bias as it is unclear to us whether the researchers could influence the choice of patients to be matched.
